# Study protocol for an observational panel study of heat strain in the general adult population in Basse Santa Su, The Gambia

**DOI:** 10.1371/journal.pone.0332238

**Published:** 2025-09-18

**Authors:** Elisabeth Tadiri, Apolline Saucy, Ana Bonell, Philip Musa Sarjo, Bakary Sonko, Jonathan Vicente dos Santos Ferreira, Moritz Burger, Moritz Gubler, Kelton Minor, Ana M. Vicedo-Cabrera

**Affiliations:** 1 Institute of Social and Preventive Medicine, University of Bern, Bern, Switzerland; 2 Oeschger Centre for Climate Change Research, University of Bern, Bern, Switzerland; 3 Medical Research Council Unit The Gambia at the London School of Hygiene & Tropical Medicine, Fajara, The Gambia; 4 Centre on Climate Change and Planetary Health, London School of Hygiene & Tropical Medicine Faculty of Epidemiology and Population Health, London, England, United Kingdom; 5 Institute of Geography, University of Bern, Bern, Switzerland; 6 Institute for Research, Development and Evaluation, Bern University of Teacher Education, Bern, Switzerland; 7 Data Science Institute, Columbia University, New York, United States of America; PLOS, UNITED KINGDOM OF GREAT BRITAIN AND NORTHERN IRELAND

## Abstract

Heat is among the most hazardous environmental factors for human health, but humidity’s role in heat-related health effects remains unclear. This study will assess the effect of humid heat and other environmental conditions on health in a representative population in Basse Santa Su, The Gambia, a region at high risk of humid heat exposure. We will examine the association between humid heat exposure and physiological heat strain, identify vulnerable sub-groups, and evaluate adaptive behaviours. We will recruit 60–90 healthy adults from Basse Santa Su and surrounding areas. Participants will be monitored for four non-consecutive weeks across dry (November–May) and rainy (June–October) seasons. Daily questionnaires will assess activities, thermal comfort, adaptation behaviours, heat strain symptoms, mood, and sleep quality. Wearables will collect time-resolved personal and indoor exposure (temperature and humidity), heat strain, and further physiological covariates. A fixed monitoring network will measure outdoor air temperature, humidity, air quality, and environmental noise. Descriptive analyses will assess baseline characteristics, heat stress and heat strain. Case-time series analysis with distributed non-linear lagged models will estimate immediate and delayed associations between exposure to humid heat and physiological heat strain. Stratified analyses by individual characteristics will explore possible vulnerability groups. Multiple exposure models and interaction terms will explore cumulative effects of multiple environmental factors. Multilinear land use regression modelling will develop high-resolution maps of temperature, humidity, and heat stress. This study will provide new insights into humid heat’s effect on health, particularly in low-income, high-exposure settings. This study addresses limitations in prior epidemiological research on heat, humidity, and health, including lack of high-resolution and individual-level data, and limited focus on humidity as a heat-health driver, on non-mortality outcomes and on climate-vulnerable populations. This study combines high-resolution microclimate mapping and individual-level measurements which may inform future epidemiological studies and heat-health interventions.

## Introduction

Extreme weather events are among the most hazardous environmental factors for human health [1]. In particular, extreme heat and associated physiological heat strain is the leading weather-related cause of death [2], accounting for over 1% of global mortality [3], comparable to mortality due to malaria, road injuries, and ozone pollution [4]. Recent extreme heat events, such as the 2022 and 2023 European heatwaves have caused thousands of excess deaths [5,6]. Heat increases mortality and morbidity across populations, but vulnerable groups (e.g., older adults, pregnant women, children) [3,7,8], those with limited adaptation capacities, and highly exposed regions [9,10] bear the greatest burden. Projections indicate a substantial rise in heat-related health impacts as temperature increases with climate change, with one in three heat-related deaths today already attributable to anthropogenic warming [11].

While the health risks of heat are well established [8,12–14], humidity’s role remains unclear. On one side, physiological models emphasize the dangers of humid heat due to impaired evaporative cooling [14]. Environmental heat stress conditions occur when the body’s cooling mechanisms are overwhelmed [15], triggering dangerous physiological responses (heat strain) that can lead to organ damage and eventually death [16,17]. For this reason, heat stress indices, integrating temperature, humidity, and weather variables, are widely used in occupational medicine (e.g., Wet Bulb Globe Temperature (WGBT)) and heat warning systems. Climate models similarly use humid heat to identify regions at risk of extreme heat stress and project future exposure to “deadly heat” under warming scenarios [9,10]. However, climate models often oversimplify vulnerability by assuming uniform temperature thresholds for health risks, conflicting with epidemiological evidence. Recent research on compound climate events underscores the need to assess interactions between multiple weather drivers, including humidity, to improve projections of heat stress under climate change scenarios [18]. On the other side, epidemiological studies often report minor [18], null or even protective effects [19] of humidity on mortality [20]. Recent large-scale population-based studies found that no heat stress indicator consistently outperformed simple temperature metrics (i.e. dry bulb temperature–the usual ambient temperature) in predicting heat-related mortality [21,20], leading to a predominant focus on dry temperature in impact assessments. Thus, humidity is rarely considered in the most recent epidemiological assessments and impacts are modeled solely based on exposure to ambient, dry temperature.

Several hypotheses could explain these discrepancies. First, most of the epidemiological research focuses on mortality as the heat-related health outcome. However, mortality may not capture the full spectrum of adverse outcomes or populations affected by heat, since older adults are more vulnerable and possibly contributing more to the total mortality burden. For example, rising temperatures have also been linked to increased hospitalizations, cardiovascular [22] and mental health disorders [19,23–27]. Less-vulnerable groups including outdoor workers, pregnant women, and children have been shown to experience adverse effects not captured in mortality data [3,7,8]. Second, humidity’s effect may vary by heat intensity, as humidity affects the body mainly above 29ºC [28], so it’s less relevant at lower temperatures. Extreme humid heat (wet bulb temperature >35ºC) events are relatively rare, localized, and occur under exceptional weather, mostly impacting low- and middle-income countries where data is lacking [9,29]. Most research to date has been conducted in high-income countries, where populations have greater adaptation capacity and lower exposure to extreme humid heat [7], limiting generalizability to low- and middle-income countries (LMICs). Understanding the health effects of humid heat in LMICs is critical, given the elevated vulnerability of these populations and the increasing frequency of extreme weather events in these regions. Despite this urgency, research in LMICs remains limited due to data availability constraints. Finally, conventional ecological study designs which average health outcomes over the population may be insufficient to capture the full range of effects, necessitating more sophisticated approaches, such as individual-level assessments. Advances in wearable sensors and personal exposure monitoring offer opportunities to reduce misclassification, improve precision, and deepen understanding of heat stress impacts beyond occupational settings, ultimately guiding more targeted public health interventions.

This study contributes to clarifying the role of humidity on heat-health impacts by focusing on a low-income setting with populations experiencing extreme and variable humid heat conditions. By incorporating individual-level data, we seek to overcome the methodological limitations of prior population-based research, allowing for a more precise evaluation of the independent effects of heat and humidity. We will also monitor intermediate physiological heat strain indicators, which may provide a more sensitive measure of humid heat effects than mortality. We will assess other environmental hazards including air pollution and noise, both as potential confounders and as independent stressors. This comprehensive approach will provide a more nuanced understanding of heat stress and its broader health implications, particularly in vulnerable populations with limited adaptation capacity.

## Materials and methods

### Aims, objectives, and research questions

The primary aim of this study is to comprehensively assess the effect of extreme humid heat and further environmental conditions on the health of the general adult population in Basse Santa Su, The Gambia, a region at high risk of extreme heat stress and humid heat exposure. The study aims to address the following key questions:

- What is the population’s exposure to ambient heat, humidity, and heat stress?
- What level of physiological heat strain is experienced by the general adult population in Basse Santa Su, and does it vary depending on the level of humidity?
- How does ambient heat stress affect physical and mental well-being, sleep, thermal sensation, comfort, and experience? Does this effect differ depending on the level of humidity?
- What is the temporal and spatial distribution of heat and heat stress and do geographical, topographical, or ecological factors influence measured heat and heat stress?
- How well do ambient monitors (both indoor and outdoor), spatially resolved data and individual measurements agree in terms of level of exposure and derived/predicted heat strain?

These research questions will be addressed through the following specific objectives:

1 Derive high-resolution, spatiotemporal maps of air temperature, humidity, and heat stress indices across the area of Basse Santa Su using onsite observations from monitors, ad hoc measurement campaigns, and geospatial data on land use and urban features.
  a Identify areas most affected by heat stress and its potential relationship with urban features.
  b Assess potential differential patterns in spatial distribution of exposure between dry and rainy seasons.
  c Assign individual exposure to temperature, humidity and various heat stress indices at the residence and work site and compare with the measurements obtained from personal devices.

We will also assign individual exposure to air pollutants and environmental noise using data from an ad hoc monitoring campaign with home indoor and outdoor monitors. However, we will not derive a spatiotemporal model and rely instead on exposure assessment methods (i.e., average level by buffer size).

2 Quantify the level of heat stress and heat strain among the population through personal measurements using wearable devices and thermo-physiological modelling.
  a Explore differences in heat stress and physiological heat strain across individual characteristics and daily activity patterns.
  b Assess agreement between different metrics (e.g., ambient monitors, spatially resolved data and personal measurements) of level of exposure and derived heat strain.
  c Determine physiological responses (heart rate, heart rate variability, skin temperature, sleep) to the thermal conditions.
  d Evaluate the individual and combined effects of environmental heat stress, air pollution, and noise on measured physiological heat strain and sleep quality.
  e Estimate the effect of environmental heat stress on daily self-reported heat sensation and experience, climate adaptation, daily activities, and physiological and mental well-being.
  f Investigate potential confounding and effect modification of the association between environmental heat stress and physiological heat strain by air pollution, noise, and sociodemographic characteristics.

### Study setting

#### Setting and design.

We will conduct an observational panel study of a representative sample of the healthy adult population living and working in Basse Santa Su and surrounding communities in The Gambia. The Gambia is a small, low-income West-African country (GDP per capita 888.20 USD [2023]) [30], largely surrounded by Senegal except for the western coastline, with a population of approximately 2.7 million, and life expectancy of 63 years [31,32]. The Gambia has a combination of tropical savannah and hot semi-arid climate (As/Av and BSh on the Köppen-Geiger climate classification system), with distinct dry (November-May) and rainy (June-October) seasons [33]. Basse Santa Su, the largest town in the Upper River Region (approximately 20,000 inhabitants [34].), is situated 6-hours inland from the coast and capital city of Banjul, and experiences high heat and humidity levels for the county, with monthly maximum temperatures exceeding 40ºC [35]. Thus, it represents a high-risk scenario for the impacts of climate change commonly seen in LMICs, as it faces considerable exposure to climate hazards, and extreme vulnerability due to limited resources for adaptation.

The cohort will be followed for one year to capture exposure across both the dry and rainy seasons. The study will consist of two main components: (1) individual monitoring of exposure, physiological heat stress, and individual characteristics to assess direct health impacts, and (2) environmental assessment and spatial mapping of humid heat and heat stress across the catchment area to capture broader exposure patterns and potential modulators of the exposure.

This study is conducted in collaboration with the Medical Research Council Unit The Gambia at the London School of Hygiene & Tropical Medicine (MRCG@LSHTM). We will use personnel, resources and infrastructure provided by the MRCG@LSHTM upon service agreement and will benefit from the established community engagement of MRCG@LSHTM.

#### Study population.

The study population will consist of 60 to 90 healthy adults from 11 communities in Basse Santa Su and the surrounding area. Eligible participants must be 18–65 years old, reside in the area for the study duration, provide written informed consent, have all four limbs intact, and communicate sufficiently in one of the study languages (Serahule, Mandinka, Fula, Wollof, or English). To avoid overlap with a parallel study of the effects of heat on pregnant women in the same region, pregnant women will be excluded, as well as those acutely unwell at recruitment.

#### Study timeline.

The study spans 18 months, beginning with a pilot phase in September 2024. Main data collection begins December 2024 for a period of 12 months, or until the target sample size with complete data is reached, with a 3–4-week buffer to account for delays. Outdoor environmental monitoring via a fixed network will capture time-resolved climate and environmental conditions continuously throughout the study.

### Patient and public involvement

The public were not directly involved in the development of the research questions, study design, or outcome measures. The public’s feedback regarding the proposed study will be sought through community sensitization.

### Recruitment

#### Community sensitization.

Community sensitization will be carried out in each community to introduce the planned study activities with the community leaders and any interested members of the community. Study staff will make a request to the alkalo (community leader) for permission for the community to take part in the study. Study staff will discuss with each community the history of MRCG@LSHTM, a locally engaged and trusted organization, introduce the proposed study in detail, address any questions, concerns, or misconceptions around the required procedures, and seek consent to conduct the study in that community.

#### Sampling.

Once community consent is obtained, eligible participants will be selected through the Basse Health and Demographic Surveillance System (HDSS), supplemented with small-scale household enumeration of nearby additional villages not included in the HDSS. Stratified random sampling by age, sex, village, and ethnicity will ensure representation of these characteristics proportional to the population.

#### Informed consent.

Potential participants will receive information sheets and study staff will conduct the written informed consent procedure in the participant’s preferred language. If the participant is unable to write, their fingerprint will be used as substitute for a signature, and an impartial adult witness will provide their signature. Potential participants will be given sufficient time to consider participation and discuss the study activities with the study staff. Participants may withdraw consent at any time during the study without consequences and their data will be deleted.

#### Enrolment and baseline data collection.

Sampled individuals will be invited for enrolment and screened for eligibility. Informed consent will be obtained from those willing to participate. Study staff will then administer a structured baseline questionnaire consisting of demographics, occupational history, housing conditions, medical history, mental well-being, physical activity, and lifestyle factors. Questions on climate adaptation behaviours, climate health literacy, and noise annoyance will also be collected. Participants will undergo a brief physical exam (height, weight, resting heart rate, and blood pressure). GPS coordinates of the household and primary workplace will be recorded for precise exposure assessment.

### Participant monitoring

**Fig 1:**
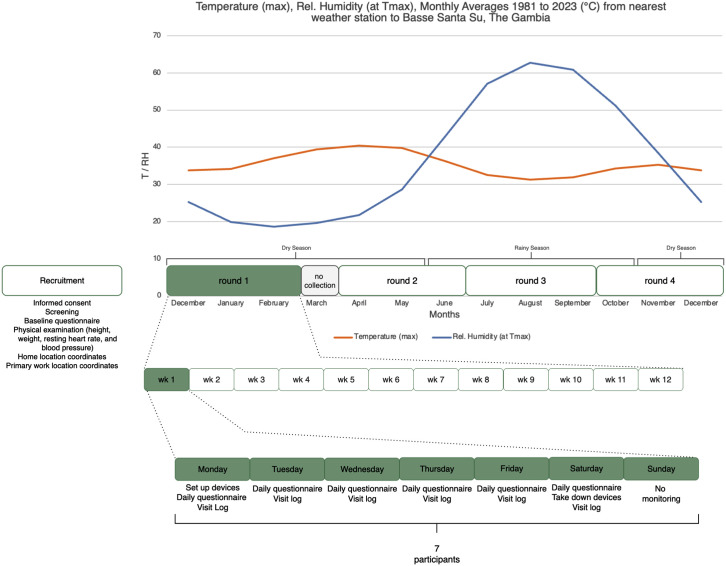
Overview of the anticipated data collection strategy throughout the year, with temperature and relative humidity historical averages from nearest weather stations. [35] Information captured from each data collection tool is described in S1 Table.

Participants will be monitored for four non-consecutive weeks throughout the study year to capture variations between the dry and rainy seasons. Each monitoring week will run Monday to Saturday (six days) (Fig 1). With seven participants monitored weekly, the minimum target sample size of 60 (each with four rounds of monitoring) can be achieved in 35 weeks assuming no loss to follow-up. Data collection may pause during Ramadan (March 2025). The maximum sample size is 90 individuals.

#### Daily questionnaire data collection.

**Fig 2:**
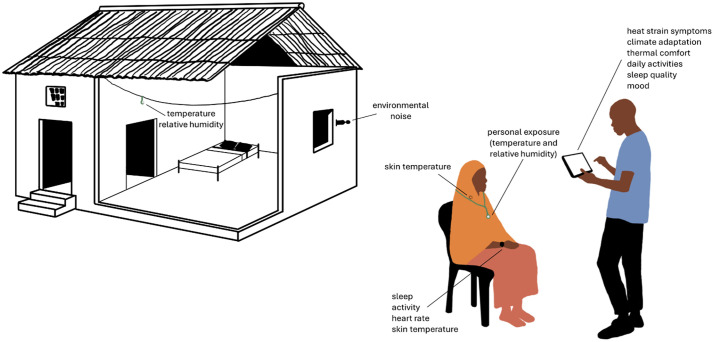
Visual representation of the individual data collection.

Each monitoring week, participants will complete daily structured questionnaires assessing activities, thermal comfort, climate adaptation behaviours, symptoms of physiological heat strain, mood, and subjective sleep quality (Fig 2). If participants become unwell during monitoring, participation will be delayed, and the study team will facilitate access to the closest routine healthcare services available. In case of heatstroke during monitoring, study staff will facilitate immediate emergency care.

#### Physiological monitoring.

Participants will be equipped with an ActiGraph LEAP [36] wristwatch, a US FDA 510(k)-cleared device designed for high-resolution digital health monitoring, to be worn continuously on the non-dominant wrist. This device includes a 3-axis accelerometer, multi-wavelength photoplethysmography PPG, gyroscope, and skin temperature sensor to continuously record physiological parameters, including heart rate, heart rate variability, step count, estimated energy expenditure, sleep onset and duration, and skin temperature. Small non-invasive temperature loggers (iButtons [37]) will be taped on participants’ clavicle using medical-grade tape to measure time-resolved skin temperature. Skin temperature measurements from the proximal and distal locations will be used to calculate weighted mean skin temperature as a proxy measure for core temperature. Physiological monitoring devices are described in detail in S2 Table.

#### Personal and household exposure monitoring.

**Fig 3:**
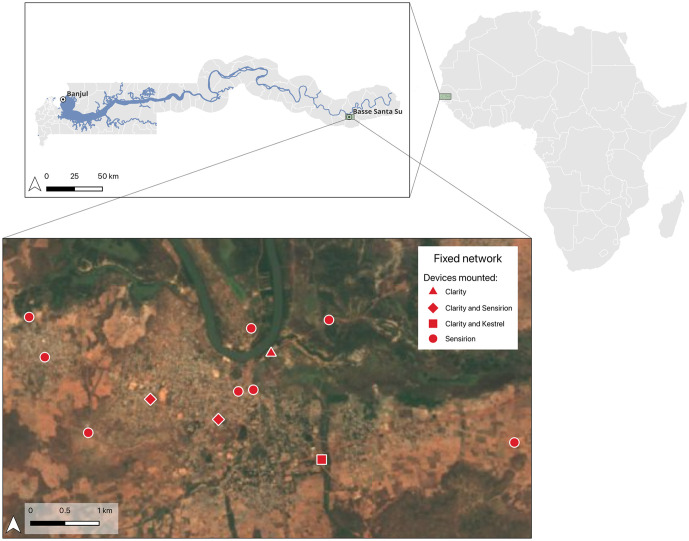
Overview of The Gambia and the study area, covering the town of Basse Santa Su (urban area) and surrounding neighbourhoods. [38–43] Expected locations of fixed monitoring stations are displayed as red points.

Individual environmental exposure monitoring will include indoor and outdoor household conditions, as well as personal exposure (Fig 3). Indoor air temperature and relative humidity will be recorded during monitoring weeks using iButton loggers [44] placed in participants’ homes. Personal exposure to temperature and relative humidity will be tracked with a wearable iButton logger [44], worn on a lanyard continuously throughout monitoring weeks. Time-resolved environmental noise will be monitored at participants’ homes with Sound Level Meter Data Loggers (NSRT_mk4) from Convergence Instruments [45],mounted on participants’ homes ideally near the bedroom window, and facing the street.

### Environmental monitoring

#### Fixed environmental monitoring network.

A fixed network of environmental monitoring stations deployed across the study area will provide continuous measurement throughout the year for micro-climate mapping (Fig 3). Sites will be selected to ensure representativeness of different environmental conditions [46]. Devices will be maintained and checked regularly to minimize possible data loss.

Low-cost Sensirion SHT4x Smart Gadget loggers [47] will be placed at fixed stations to monitor temperature and relative humidity at a 10-minute resolution. These will be coupled with low-cost, custom-made, passively ventilated radiation shields used in previous studies [46]. Air temperature, globe temperature, relative humidity, wind, as well as device-calculated measures such as wet bulb globe temperature (WBGT) will be measured with Kestrel 5400 Heat Stress Monitors [48] in at least one representative site.

Fixed stations will measure PM2.5 and NO_2_ every 15 minutes using the Clarity Node-S [49] Attached wind modules will collect wind speed, wind direction, temperature, relative humidity, and barometric pressure. These stations will be calibrated on-site and serve as reference stations for the network. Environmental noise will be measured for at least two weeks at a time at each fixed station during both the dry and rainy seasons using NSRT_mk4 Sound Level Meter Data Loggers [45]. A full device list is in S2 Table.

#### Meteorological and remote-sensing data.

Additional spatially resolved data will be sourced to derive maps of heat stress for the study site using land use regression modelling. These include meteorological reanalysis data (air temperature, humidity, solar radiation, wind speed and direction, and atmospheric pressure) (ERA5-Land, Copernicus, 8 km resolution) and remote-sensing data providing high-resolution environmental variables (ESRI/ESA land cover data (10 m resolution), topography (digital elevation model; NASA: 30m resolution); vegetation (leaf area index; GCOM-C/SGLI L3: 30m resolution); and urban surface geometry (World Settlement Footprint; DLR: 10m)).

### Statistical analyses

#### Land use regression modelling.

We will use measurements from monitors, ad hoc measurement campaigns, and geospatial sources to derive high-resolution, spatiotemporal maps of temperature, humidity, and heat stress indices across Basse Santa Su. Multilinear landuse regression (LUR) models will estimate high spatial and temporal resolution heat and heat stress distribution across the study area using a supervised stepwise procedure (hybrid version of forward and backward) to maximize the percentage of explained variability (R^2^). Predictors will include measured weather variables from the ad hoc monitoring campaign and data from global high-resolution products. The resulting predictive models will be used to calculate single weather variables and combined indices (e.g. wet bulb temperature (WBT), WBGT, physiological equivalent temperature (PET), Universal Thermal Climate Index (UTCI)) in each 50x50-meter cell. These can be estimated from single measurements of mean temperature, relative humidity, atmospheric pressure, wind speed and mean radiant temperature using available algorithms. Models will be validated using leave-one-out cross validation procedure. We will map the spatial and temporal variation of these variables across the study region and identify high-risk neighbourhoods, contextual variables, and periods of the year associated with elevated or reduced exposure to heat stress.

#### Assessing the risk of humid heat strain in a high-risk population.

We will assess the effect of humid heat and further environmental conditions on health in the sample population by combining land use model data, monitor data and individual data (questionnaires and wearables). Time-resolved heat strain (physiological strain index (PSI)) will be calculated using information from the wearable devices. PSI is a widely accepted metric for assessing physiological heat strain that goes from 0 (no strain) to 10 (very high strain) and can be computed easily using heart rate (HR) and core temperature (CT) measurements. The standard method for measuring CT is through rectal thermistors, which is not suitable in the study setting. Thus, we will use validated algorithms to compute CT from wearable-recorded skin temperature. Self-reported heat strain symptoms will help evaluate the estimated physiological heat strain.

Descriptive analyses will assess baseline characteristics, heat stress (i.e., individual measurement, exposure from microclimate assessment), heat strain (PSI), noise and air pollution. Linear, non-linear, and logistic mixed models will assess the association between time-resolved humid heat exposure and physiological heat strain. Stratified analyses will assess the association by sociodemographic characteristics, daily activities, and levels of air pollution and noise. Analyses will be adjusted for time-varying confounders (e.g. time-resolved measured, modelled noise and air pollution).

Secondary health outcomes (e.g. sleep quality, mental well-being) will be determined from the wearable devices and questionnaires, respectively. Likewise, linear, non-linear, and logistic mixed models will assess the association between exposure to humid heat and secondary health outcomes. Similar models will be developed to estimate the association between heat strain, and air pollution and noise. Stratified analyses will estimate effect modification by individual and environmental characteristics and activity type.

#### Handling of missing data.

We will use inverse probability weighting methods to correct for possible loss to follow-up and missing data, and multiple imputation methods for mixed-effects models (e.g. R MICE package [50]) for missing questionnaire data. Missing environmental data can be imputed using simultaneous measurement data from nearby fixed network sites.

#### Sample Size.

Due to a lack of literature on the study topic, a formal sample size estimation was not possible, but was informed by comparable studies [51,52]. A minimum sample size of 60 participants has been estimated, with approximately 30% oversampling to compensate for expected loss to follow-up, for a total target enrolment of 77 participants in all four rounds of data collection. Enrolment began on 18 November 2024 and may continue throughout the data collection up to a maximum of 90 participants should dropouts occur in subsequent rounds of data collection. Data collection is estimated to be completed by the end of 2025, with initial results expected by mid-2026. The case-time series design allows participants to serve as their own controls in panel studies, with the advantage of avoiding bias from unmeasured, time-invariant variables such as individual sociodemographic characteristics. With this design, power is not only defined by the number of individuals, but mostly by the number of repeated measurements in each participant, as well as the within-individual exposure and outcome variability. Assuming a one-hour temporal resolution, each of the four monitoring periods will yield approximately 11,000 repeated data points (44,000 datapoints for the study period). Comparatively, one study conducted in Burkina Faso collected wearable data on 143 participants for a year and successfully linked climate variables with changes in daily sleep and daily activities, similar to our secondary objectives [53]. In contrast, our primary objective uses time-resolved variables (e.g. one hour resolution) and precise climate exposure data, increasing power of the study.

### Bias and mitigation

Potential for recall bias introduced in the participant questionnaires will be assessed by piloting the questionnaires, and mitigated by reducing the recall period through timely administration. Potential social desirability bias with any sensitive questions (e.g., questions around drug and alcohol use, or mental well-being) will be mitigated by using trained fieldworkers from the MRCG@LSHTM, and by informing participants that all responses are confidential, and data will be anonymized.

Environmental measurement error will be mitigated through testing and calibration of devices. Field testing of new devices prior to the start of the study showed a very high level of agreement between devices on temperature and relative humidity, and devices will be co-located with a reference monitor at end of the study. For air pollution measurements, no reference monitor is available for co-location. However, Clarity Node-S devices are pre-calibrated with a standard global calibration procedure. Further, a combination of modelled, and home indoor and outdoor data collection will reduce bias due to exposure misclassification. Spatial climate and environmental models will be validated against individual measurements.

### Ethical considerations

This study will be conducted in accordance with the protocol, the Declaration of Helsinki [54], the principles of Good Clinical Practice, the Human Research Act (HRA) and the Human Research Ordinance (HRO) [55] as well as other locally relevant regulations.

Ethical approval was received from Gambia Government/MRCG@LSHTM Joint Ethics Committee (29838 approved 05 December 2023), London School of Hygiene & Tropical Medicine Observational Research Ethics Committee (29838 approved 30 January 2024) and from the Kantonale Ethikkommission Bern (2024−01167 approved 19 July 2024).

All participants will provide written informed consent. No biological samples will be collected. Data collection is non-invasive and minimally time-consuming. Survey data will be collected on password-protected tablets using REDCap electronic data capture, hosted at MRCG@LSHTM and accessed by the University of Bern [56,57]. Participants will be assigned unique identifying numbers (UINs) for all data collection forms and databases, and data will be stored on encrypted, password protected devices with access restricted to study investigators. Identifiable data will not be shared or published. Further details on data management are described in S1 File.

## Discussion

This study will fill critical gaps in the research on the health effects of humid heat, and in general, on the impacts of climate and climate change on human health. Primarily, this study will provide new insights into the role of humidity on heat-health impacts using real-world physiological data in a real extreme environment rather than in a lab environment. Individual-level data and in situ environmental measurements will better capture heat strain and heat stress, enabling evaluation of the interaction between humidity and heat and how it affects physiological strain in individuals exposed to extreme weather conditions. This research will also provide critical evidence for The Gambia and similar low-resource settings, which are projected to be some of the most vulnerable to the impacts of climate change and extreme weather conditions [58]. Upon project completion, results will be shared with participating communities through a local dissemination event co-organised with the MRCG@LSHTM, ensuring culturally appropriate setting and languages. Results will also be presented at local and international conferences, to key stakeholders, and submitted to open-access peer-reviewed journals. Findings from this study may have important implications for health policy and climate adaptation strategies, especially in LMICs with high exposure to heat stress, and could aid in development of interventions to mitigate impacts of extreme climate (e.g. heat health warning systems, urbanization plans and adaptation plans).

## Supporting information

S1 Table Data collection tools. (DOCX)

S2 Table Overview of personal and environmental monitoring devices. (DOCX)

S1 File Data management. (DOCX)

S2 Inclusivity-in-global-research-questionnaire_19.08.2025. (DOCX)

## References

[1] IPCC, Press CU. Climate Change 2021: The Physical Science Basis. Contribution of Working Group I to the Sixth Assessment Report of the Intergovernmental Panel on Climate Change. (Masson-Delmotte V; PZAPSLCCPSBNCYCLGMIGMHKLELJBRMTKMTWOYRYBZ, ed.). IPCC; 2021. https://www.ipcc.ch/report/ar6/wg1/#FullReport

[2] Romanello M, Di Napoli C, Drummond P, Green C, Kennard H, Lampard P, et al. The 2022 report of the Lancet Countdown on health and climate change: health at the mercy of fossil fuels. Lancet. 2022;400(10363):1619–54. doi: 10.1016/S0140-6736(22)01540-9 36306815 PMC7616806

[3] Zhao Q, Guo Y, Ye T, Gasparrini A, Tong S, Overcenco A, et al. Global, regional, and national burden of mortality associated with non-optimal ambient temperatures from 2000 to 2019: a three-stage modelling study. Lancet Planet Health. 2021;5(7):e415–25. doi: 10.1016/S2542-5196(21)00081-4 34245712

[4] D GB. Global burden of diseases. 2023. https://www.healthdata.org/research-analysis/gbd

[5] Ballester J, Quijal-Zamorano M, Méndez Turrubiates RF, Pegenaute F, Herrmann FR, Robine JM, et al. Heat-related mortality in Europe during the summer of 2022. Nat Med. 2023;29(7):1857–66. doi: 10.1038/s41591-023-02419-z 37429922 PMC10353926

[6] Gallo E, Quijal-Zamorano M, Méndez Turrubiates RF, Tonne C, Basagaña X, Achebak H, et al. Heat-related mortality in Europe during 2023 and the role of adaptation in protecting health. Nat Med. 2024;30(11):3101–5. doi: 10.1038/s41591-024-03186-1 39134730

[7] Green H, Bailey J, Schwarz L, Vanos J, Ebi K, Benmarhnia T. Impact of heat on mortality and morbidity in low and middle income countries: A review of the epidemiological evidence and considerations for future research. Environ Res. 2019;171:80–91. doi: 10.1016/j.envres.2019.01.010 30660921

[8] Guo Y, Gasparrini A, Armstrong BG, Tawatsupa B, Tobias A, Lavigne E, et al. Heat Wave and Mortality: A Multicountry, Multicommunity Study. Environ Health Perspect. 2017;125(8):087006. doi: 10.1289/EHP1026 28886602 PMC5783630

[9] Raymond C, Matthews T, Horton RM, Fischer EM, Fueglistaler S, Ivanovich C, et al. On the Controlling Factors for Globally Extreme Humid Heat. Geophysical Research Letters. 2021;48(23). doi: 10.1029/2021gl096082

[10] Mora C, Dousset B, Caldwell IR, Powell FE, Geronimo RC, Bielecki CR, et al. Global risk of deadly heat. Nature Clim Change. 2017;7(7):501–6. doi: 10.1038/nclimate3322

[11] Vicedo-Cabrera AM, Scovronick N, Sera F, Royé D, Schneider R, Tobias A, et al. The burden of heat-related mortality attributable to recent human-induced climate change. Nat Clim Chang. 2021;11(6):492–500. doi: 10.1038/s41558-021-01058-x 34221128 PMC7611104

[12] Gasparrini A, Guo Y, Hashizume M, Lavigne E, Zanobetti A, Schwartz J, et al. Mortality risk attributable to high and low ambient temperature: a multicountry observational study. Lancet. 2015;386(9991):369–75. doi: 10.1016/S0140-6736(14)62114-0 26003380 PMC4521077

[13] Anderson GB, Bell ML. Heat waves in the United States: mortality risk during heat waves and effect modification by heat wave characteristics in 43 U.S. communities. Environ Health Perspect. 2011;119(2):210–8. doi: 10.1289/ehp.1002313 21084239 PMC3040608

[14] Gronlund CJ, Sullivan KP, Kefelegn Y, Cameron L, O’Neill MS. Climate change and temperature extremes: A review of heat- and cold-related morbidity and mortality concerns of municipalities. Maturitas. 2018;114:54–9. doi: 10.1016/j.maturitas.2018.06.002 29907247 PMC6754702

[15] Ioannou LG, Mantzios K, Tsoutsoubi L, Notley SR, Dinas PC, Brearley M, et al. Indicators to assess physiological heat strain - Part 1: Systematic review. Temperature (Austin). 2022;9(3):227–62. doi: 10.1080/23328940.2022.2037376 36211945 PMC9542768

[16] Mora C, Counsell CWW, Bielecki CR, Louis LV. Twenty-Seven Ways a Heat Wave Can Kill You: Deadly Heat in the Era of Climate Change. Circ Cardiovasc Qual Outcomes. 2017;10(11):e004233. doi: 10.1161/CIRCOUTCOMES.117.004233 29122837

[17] Buzan JR, Huber M. Moist Heat Stress on a Hotter Earth. Annu Rev Earth Planet Sci. 2020;48(1):623–55. doi: 10.1146/annurev-earth-053018-060100

[18] Barreca AI. Climate change, humidity, and mortality in the United States. J Environ Econ Manage. 2012;63(1):19–34. doi: 10.1016/j.jeem.2011.07.004 25328254 PMC4199665

[19] Vicedo-Cabrera AM, Melén E, Forastiere F, Gehring U, Katsouyanni K, Yorgancioglu A, et al. Climate change and respiratory health: a European Respiratory Society position statement. Eur Respir J. 2023;62(2):2201960. doi: 10.1183/13993003.01960-2022 37661094

[20] Barnett AG, Tong S, Clements ACA. What measure of temperature is the best predictor of mortality?. Environ Res. 2010;110(6):604–11. doi: 10.1016/j.envres.2010.05.006 20519131

[21] Rodopoulou S, Samoli E, Analitis A, Atkinson RW, de’Donato FK, Katsouyanni K. Searching for the best modeling specification for assessing the effects of temperature and humidity on health: a time series analysis in three European cities. Int J Biometeorol. 2015;59(11):1585–96. doi: 10.1007/s00484-015-0965-2 25638489

[22] Requia WJ, Alahmad B, Schwartz JD, Koutrakis P. Association of low and high ambient temperature with mortality for cardiorespiratory diseases in Brazil. Environ Res. 2023;234:116532. doi: 10.1016/j.envres.2023.116532 37394170

[23] Kim Y, Kim H, Gasparrini A, Armstrong B, Honda Y, Chung Y, et al. Suicide and Ambient Temperature: A Multi-Country Multi-City Study. Environ Health Perspect. 2019;127(11):117007. doi: 10.1289/EHP4898 31769300 PMC6927501

[24] Carleton TA. Crop-damaging temperatures increase suicide rates in India. Proc Natl Acad Sci U S A. 2017;114(33):8746–51. doi: 10.1073/pnas.1701354114 28760983 PMC5565417

[25] Page LA, Hajat S, Kovats RS. Relationship between daily suicide counts and temperature in England and Wales. Br J Psychiatry. 2007;191:106–12. doi: 10.1192/bjp.bp.106.031948 17666493

[26] Bär S, Bundo M, de Schrijver E, Müller TJ, Vicedo-Cabrera AM. Suicides and ambient temperature in Switzerland: A nationwide time-series analysis. Swiss Med Wkly. 2022;152:w30115. doi: 10.4414/smw.2022.w30115 35262317

[27] Thompson R, Lawrance EL, Roberts LF, Grailey K, Ashrafian H, Maheswaran H, et al. Ambient temperature and mental health: a systematic review and meta-analysis. Lancet Planet Health. 2023;7(7):e580–9. doi: 10.1016/S2542-5196(23)00104-3 37437999

[28] Brunt D. The reactions of the human body to its physical enviroment. Quart J Royal Meteoro Soc. 1943;69(300):77–114. doi: 10.1002/qj.49706930002

[29] Raymond C, Matthews T, Horton RM. The emergence of heat and humidity too severe for human tolerance. Sci Adv. 2020;6(19):eaaw1838. doi: 10.1126/sciadv.aaw1838 32494693 PMC7209987

[30] GDP per capita (current US$) - Gambia, The | Data. Cited April 14, 2025. https://data.worldbank.org/indicator/NY.GDP.PCAP.CD?end=2023&locations=GM&start=1966&view=chart

[31] Population, total - Gambia, The | Data. Cited March 18, 2025. https://data.worldbank.org/indicator/SP.POP.TOTL?locations=GM

[32] Life expectancy at birth, total (years) - Gambia, The | Data. Cited March 18, 2025. https://data.worldbank.org/indicator/SP.DYN.LE00.IN?locations=GM

[33] Gambia, The - Summary | Climate Change Knowledge Portal. Cited April 14, 2025. https://climateknowledgeportal.worldbank.org/country/gambia

[34] Gambia, The Cities Database | Simplemaps.com. Cited April 14, 2025. https://simplemaps.com/data/gm-cities

[35] Your Area: Today (switch to: Tomorrow or WorkHeat) | Climate CHIP. Cited February 13, 2025. https://climatechip.org/your-area-climate-data

[36] ActiGraph LEAP | ActiGraph Wearable Devices. Cited February 16, 2025. https://theactigraph.com/actigraph-leap

[37] Thermochron | Temperature Logging iButtons. Cited May 16, 2025. https://www.ibuttonlink.com/collections/thermochron

[38] Runfola D, Anderson A, Baier H, Crittenden M, Dowker E, Fuhrig S, et al. geoBoundaries: A global database of political administrative boundaries. PLoS One. 2020;15(4):e0231866. doi: 10.1371/journal.pone.0231866 32330167 PMC7182183

[39] Gambia Waterways (OpenStreetMap Export) | Humanitarian Dataset | HDX. Cited April 16, 2025. https://data.humdata.org/dataset/hotosm_gmb_waterways

[40] Africa Countries | ArcGIS Hub. Cited April 16, 2025. https://hub.arcgis.com/datasets/africa::africa-countries/about

[41] Node: Banjul (249167555) | OpenStreetMap. Cited April 16, 2025. https://www.openstreetmap.org/node/249167555#map=19/13.455350/-16.575646&layers=D

[42] Node: Basse Santa Su (2612325231) | OpenStreetMap. Cited April 16, 2025. https://www.openstreetmap.org/node/2612325231#map=11/13.2927/-14.2592&layers=D

[43] EOX::Maps - Sentinel-2 cloudless 2018. Sentinel-2 cloudless - https://s2maps.eu by EOX IT Services GmbH (Contains modified Copernicus Sentinel data 2016 & 2017). Licensed under a Creative Commons Attribution 4.0 International License. 2018. Accessed September 1, 2025. https://qms.nextgis.com/geoservices/2173/

[44] Humidity Logging iButtons. Cited May 16, 2025. https://www.ibuttonlink.com/collections/humidity-logging-ibuttons

[45] NSRT_mk4 Sound Level Meter with Type 1 Microphone and Data Logger. Cited May 16, 2025. https://convergenceinstruments.com/product/sound-level-meter-data-logger-with-type-1-microphone-nsrt_mk4/?srsltid=AfmBOoo2b763-I3n7qzs1c5ku40GXn44F3_0nkSE4-qw0LIHABbFQIy8

[46] Gubler M, Christen A, Remund J, Brönnimann S. Evaluation and application of a low-cost measurement network to study intra-urban temperature differences during summer 2018 in Bern, Switzerland. Urban Climate. 2021;37:100817. doi: 10.1016/j.uclim.2021.100817

[47] SHT4x Smart Gadget - SHT4x Humidity and Temperature Sensor Demonstrator. Cited May 16, 2025. https://sensirion.com/products/catalog/SHT4x-Smart-Gadget

[48] Kestrel 5400 HST Heat Stress Tracker, Weather & WBGT Meter. Cited May 16, 2025. https://kestrelinstruments.com/kestrel-5400-heat-stress-tracker?srsltid=AfmBOoo_d6pIjXc6nmcmDBk6z7VrE-ZiIvIKA5YsUrcmvEvok5aUntAi

[49] Clarity Node-S: PM & NO₂ Air Quality Sensor. Cited May 16, 2025. https://www.clarity.io/products/clarity-node-s?utm_term=&utm_campaign=Competitor+Pmax&utm_source=adwords&utm_medium=ppc&hsa_acc=1787529554&hsa_cam=22041995485&hsa_grp=&hsa_ad=&hsa_src=x&hsa_tgt=&hsa_kw=&hsa_mt=&hsa_net=adwords&hsa_ver=3&gad_source=1&gad_campaignid=22239415138&gbraid=0AAAAACPUJUO1St43t-5UpGPBhWOTfyqz8&gclid=Cj0KCQjwxJvBBhDuARIsAGUgNfiDEK346n7kAUY-sw8MIrORbQtKPwTZwICc9oZRxLQkCVbVIQL3KEIaApnQEALw_wcB

[50] Buuren S van, Groothuis-Oudshoorn K. mice: Multivariate Imputation by Chained Equations inR. J Stat Soft. 2011;45(3). doi: 10.18637/jss.v045.i03

[51] Runkle JD, Cui C, Fuhrmann C, Stevens S, Del Pinal J, Sugg MM. Evaluation of wearable sensors for physiologic monitoring of individually experienced temperatures in outdoor workers in southeastern U.S. Environ Int. 2019;129:229–38. doi: 10.1016/j.envint.2019.05.026 31146157

[52] Milà C, Curto A, Dimitrova A, Sreekanth V, Kinra S, Marshall JD, et al. Identifying predictors of personal exposure to air temperature in peri-urban India. Sci Total Environ. 2020;707:136114. doi: 10.1016/j.scitotenv.2019.136114 31863998

[53] Koch M, Matzke I, Huhn S, Sié A, Boudo V, Compaoré G, et al. Assessing the Effect of Extreme Weather on Population Health Using Consumer-Grade Wearables in Rural Burkina Faso: Observational Panel Study. JMIR Mhealth Uhealth. 2023;11:e46980. doi: 10.2196/46980 37938879 PMC10666008

[54] WMA Declaration of Helsinki – Ethical Principles for Medical Research Involving Human Subjects – WMA – The World Medical Association. Cited May 24, 2024. https://www.wma.net/policies-post/wma-declaration-of-helsinki-ethical-principles-for-medical-research-involving-human-subjects/

[55] SR 810. 301 - Ordinance of 20 September 2013 on H . . . | Fedlex. Cited May 24, 2024. https://www.fedlex.admin.ch/eli/cc/2013/642/en

[56] Harris PA, Taylor R, Minor BL, Elliott V, Fernandez M, O’Neal L, et al. The REDCap consortium: Building an international community of software platform partners. J Biomed Inform. 2019;95:103208. doi: 10.1016/j.jbi.2019.103208 31078660 PMC7254481

[57] Harris PA, Taylor R, Thielke R, Payne J, Gonzalez N, Conde JG. Research electronic data capture (REDCap)--a metadata-driven methodology and workflow process for providing translational research informatics support. J Biomed Inform. 2009;42(2):377–81. doi: 10.1016/j.jbi.2008.08.010 18929686 PMC2700030

[58] Serdeczny O, Adams S, Baarsch F, Coumou D, Robinson A, Hare W, et al. Climate change impacts in Sub-Saharan Africa: from physical changes to their social repercussions. Reg Environ Change. 2016;17(6):1585–600. doi: 10.1007/s10113-015-0910-2

