# Supplementary material for: Study protocol for an observational panel study of heat strain in the general adult population in Basse Santa Su, The Gambia
Source: PLoS One. 2025 Sep 18;20(9):e0332238. doi: 10.1371/journal.pone.0332238 (PMC12445497; doi:10.1371/journal.pone.0332238)
Supplement: S1 Table — (DOCX) [file pone.0332238.s001.docx]

**S1. Table DATA COLLECTION TOOLS**

| Data collection tool | Frequency | Content |
| --- | --- | --- |
| Informed consent checklist | Once, at recruitment | Confirmation of informed consent |
| Screening checklist | Once, at recruitment | Inclusion and exclusion criteria |
| Baseline questionnaire | Once, at recruitment | Demographics Occupation Socioeconomics Brief Household Water Insecurity Experiences (HWISE-4) Scale Housing Medical History Exercise and physical activity Kessler Psychological Distress Scale (K6) Drug and alcohol use Thermal satisfaction Climate health literacy Climate adaptation Noise pollution and annoyance Air quality |
| Home GPS log | Once, at recruitment | GPS coordinates of household |
| Work GPS log | Once, at recruitment, and additionally when participant changes primary occupation | GPS coordinates of primary workplace |
| Daily questionnaire | Daily during monitoring weeks (6 days) | Thermal sensation, comfort, and experience Daily activities Heat exhaustion symptoms Heat adaptation measures Nightly sleep quality and disturbance Daily mood |
|  | First day only of monitoring weeks | Change of home or primary work location  Physical health |
|  | Last day only of monitoring weeks | Weekly sleep quality and disturbance Weekly mental wellbeing (WHO-5)  Weekly air quality, noise, and ventilation experience |
| Participant visit log | Any time individual monitoring devices are set up, taken down, or charged | Actions performed on any individual monitoring device |
| Withdrawal form | Any time a participant withdraws from the study | Reason for withdrawal |
| Fixed station log | Any time fixed station devices are set up, taken down, or charged | Current environmental conditions Actions performed on any fixed station device |
|  | At set-up only | GPS coordinates, height from ground and distance from road |
