# Supplementary material for: Study protocol for an observational panel study of heat strain in the general adult population in Basse Santa Su, The Gambia
Source: PLoS One. 2025 Sep 18;20(9):e0332238. doi: 10.1371/journal.pone.0332238 (PMC12445497; doi:10.1371/journal.pone.0332238)
Supplement: S2 Table — (DOCX) [file pone.0332238.s002.docx]

### **S2. Table OVERVIEW OF PERSONAL AND ENVIRONMENTAL MONITORING DEVICES**

| Monitoring | Device | Measurement(s) of interest | Location | Resolution |
| --- | --- | --- | --- | --- |
| Physiological | ActiGraph LEAP | Heart rate  Heart rate variability  Step count  Estimated energy expenditure  Sleep onset and duration  Skin temperature | Non-dominant wrist | 1 minute |
| Personal / household exposure | iButton (DS1921H-F5# Thermochron) | Skin temperature | Taped on clavicular skin | 15 minutes |
|  | iButton (DS1923-F5# Hygrochron) | Air temperature  Relative humidity | Lanyard around the neck | 15 minutes |
|  | Sound Level Meter Data Logger (NSRT mk4) | Environmental Noise | Outside bedroom window | 15 minutes |
|  |  |  |  |  |
| Fixed network | Clarity Node-S  Without wind module | PM_2.5_  NO_2_ | Across Basse Santa Su | 15 minutes |
|  | With wind module | PM_2.5_  NO_2_  Wind speed  Wind direction  Air temperature  Relative humidity  Barometric pressure |  |  |
|  | Sensirion SHT4x Smart Gadget | Air temperature  Relative humidity | Across Basse Santa Su | 10 minutes |
|  | NSRT_mk4 | Environmental noise | Across Basse Santa Su | 15 minutes |
|  | Kestrel 5400 | Wind Speed  Air temperature  Globe temperature  Relative humidity  Pressure  Device-calculated measurements (e.g. WBGT) | Across Basse Santa Su | 60 minutes |
