# Supplementary material for: Study protocol for an observational panel study of heat strain in the general adult population in Basse Santa Su, The Gambia
Source: PLoS One. 2025 Sep 18;20(9):e0332238. doi: 10.1371/journal.pone.0332238 (PMC12445497; doi:10.1371/journal.pone.0332238)
Supplement: S1 File — (DOCX) [file pone.0332238.s003.docx]

### **S1 File. DATA MANAGEMENT**

#### **Data quality assurance**

All study staff will be trained in research ethics and protocols prior to the start of study. Data collection will follow standardized operating procedures (SOPs) to ensure quality. Data will be reviewed regularly for completeness by the MRCG@LSHTM data team and the study coordinators, and meetings will be held regularly to discuss progress and challenges.

#### **Confidentiality and ethical considerations**

Data access will be restricted to authorized personnel who require the data to fulfil their duties within the research project scope. Participants will be allocated unique identifying numbers (UIN) at recruitment. Physiological and individual environmental exposure data will be downloaded from the devices, linked to the UIN, transferred to a University of Bern server, and wiped from the devices. Survey data will be collected on password-protected tablets using REDCap electronic data capture, hosted at MRCG@LSHTM and accessed by the University of Bern.^58,59^ Each study visit day, tablets will be synced, allowing transfer of encrypted data to the designated server. Environmental data from the fixed network will be regularly transferred to the University of Bern server.

All data stored on MRCG@LSHTM severs will be backed up regularly in accordance with MRC SOP-INT-001. The database is centrally stored, and data are secure and encrypted. No personal identifiable information will be available in any shared or published document.

#### **Retention and destruction of project data**

Data will be analysed and submitted to open-access peer-reviewed journals. We will comply with international open-access standards and guidelines. All study documents will be archived for at least 10 years. Anonymised, un-linkable original data, code and results may be made available through the University of Bern’s BORIS repository.
